# Investigating human platelet antigens and antibodies in pregnancy: An 11‐year experience from a Canadian reference laboratory

**DOI:** 10.1111/vox.70210

**Published:** 2026-02-11

**Authors:** Bryan Tordon, Gwen Clarke, Jacqueline Wong, Ann Peregrin, Akash Gupta

**Affiliations:** ^1^ Department of Laboratory Medicine and Pathology University of Calgary Calgary Alberta Canada; ^2^ Laboratory Medicine and Pathology University of Alberta Edmonton Alberta Canada; ^3^ National Platelet Immunology Reference Laboratory Canadian Blood Services Brampton Ontario Canada; ^4^ Medical Affairs and Innovation Canadian Blood Services Ottawa Ontario Canada; ^5^ Department of Laboratory Medicine and Pathobiology University of Toronto Toronto Ontario Canada

**Keywords:** foetal and neonatal alloimmune thrombocytopaenia, immunology, platelets, thrombocytopaenia

## Abstract

**Background and Objectives:**

Foetal and neonatal alloimmune thrombocytopaenia (FNAIT) is a potentially severe immune‐mediated condition that is believed to be under‐identified in the Canadian population. The Canadian Blood Services National Platelet Immunology Reference Laboratory (NPIRL) serves as a centralized referral laboratory for FNAIT investigations across Canada. Diagnostic evaluation includes human platelet antigen (HPA) genotyping and maternal anti‐HPA antibody testing. This study is a review of all FNAIT investigations referred to NPIRL to assess testing patterns, antibody detection rates and evidence of under identification at a national level.

**Materials and Methods:**

All maternal, paternal and neonatal samples related to FNAIT investigations between 20 January 2014 and 31 December 2024 were extracted from the NPIRL database. The geographic origin of samples, HPA genotyping and antibody specificity, if present, were documented.

**Results:**

A total of 1986 samples (1076 maternal, 620 paternal and 290 neonatal) were received for FNAIT investigation over the 11‐year study period. One‐hundred and thirty‐five HPA antibodies were identified in 130 of the maternal samples tested, with anti‐HPA‐1a and HPA‐5b being the most frequently detected specificities. Despite a modest increase in annual referrals, the total number of investigations and antibody‐positive cases was substantially lower than expected based on Canadian birth rates and published FNAIT incidence estimates.

**Conclusion:**

Referral patterns and laboratory findings from NPIRL demonstrate a marked discrepancy between expected FNAIT incidence and observed diagnostic activity in Canada, indicating that FNAIT is substantially under‐identified. These findings highlight the need for improved recognition, standardized investigation pathways and enhanced diagnostic strategies to reduce missed cases and improve neonatal outcomes.

**Highlights**

- This study provides the national overview of foetal and neonatal alloimmune thrombocytopaenia (FNAIT) investigations from the National Platelet Immunology Reference Laboratory in Canada.
- Maternal, paternal and neonatal sample testing is used to determine maternal/neonatal incompatibility, assess clinical significance and inform perinatal management for current and future pregnancies.
- The number of investigations requested is much lower than the expected incidence of FNAIT, suggesting substantial under‐identification of FNAIT in Canada.

## INTRODUCTION

Foetal and neonatal alloimmune thrombocytopaenia (FNAIT) is a rare condition resulting from incompatibility between maternal and foetal human platelet antigen (HPA) expression due to foetal inheritance of paternal HPAs and subsequent maternal alloimmunization to those antigens. FNAIT causes an immune‐mediated thrombocytopaenia, typically affecting offspring during the second and third trimesters of pregnancy. Outcomes range from mild thrombocytopaenia to intracranial haemorrhage (ICH) and neonatal/foetal death [1]. Estimated incidence is between 1 in 1000 and 1 in 2000 live births based on published data [2, 3]. Despite this, the true frequency of FNAIT in Canada remains unknown and is likely under‐identified. While the Canadian National Advisory Committee on Blood and Blood Products [4] has endorsed the International Collaboration for Transfusion Medicine Guidelines FNAIT guidelines [5], in the absence of routine antenatal screening and standardized national investigation pathways, FNAIT is most often investigated after the onset of neonatal thrombocytopaenia or unexpected foetal/neonatal bleeding. Milder cases may resolve without recognition, while severe cases may result in intrauterine death without follow‐up investigations. Consequently, FNAIT is likely underrepresented in the current Canadian diagnostic data.

Based on Statistics Canada data, approximately 300,000 live births occur in Canada outside of Quebec annually [6]. Therefore, the expected number of FNAIT cases is likely between 200 and 300 cases annually. However, only a small fraction of this number undergoes laboratory investigation at the Canadian National Platelet Immunology Reference Laboratory (NPIRL).

Maternal antibodies directed against HPAs expressed on foetal platelets can develop and become clinically significant during a first pregnancy [3]. The most common implicated antibody specificity differs depending upon the patient's ethnic background. In White populations, anti‐HPA‐1a is most commonly implicated, followed by anti‐HPA‐5b and anti‐HPA‐3a [7]. In Asian populations, anti‐HPA‐4a and anti‐CD36 are more frequently implicated [8]. HPA incompatibility between mother and foetus is associated with a 10% chance of forming anti‐HPA‐1a antibody, whereas the alloimmunization rates against the other HPAs is currently unknown. Despite antibody development, only a fraction of HPA alloimmunized individuals go on to have a foetus or neonate impacted by FNAIT [3].

The Canadian NPIRL is located in Brampton, Ontario, and receives samples from all provinces and territories across the country, excluding the province of Quebec. This reference laboratory provides specialized testing for various clinical conditions related to anti‐platelet and anti‐human leukocyte antigen (HLA) antibodies, including FNAIT, transfusion‐related acute lung injury (TRALI) and post‐transfusion purpura, and performs HLA testing on blood donors and patients.

FNAIT investigations at NPIRL involve a combination of maternal antibody screening, HPA genotyping of maternal, paternal and neonatal samples and confirmatory testing using the monoclonal antibody–specific immobilization of platelet antigens (MAIPA) assay. Maternal serum is initially screened for anti‐HPA antibodies by one of two methods: an enzyme‐linked immunosorbent assay (ELISA), or a bead‐based assay where maternal anti‐HPA becomes bound to synthetic beads coated with platelet glycoproteins and is detected via fluorescence in flow cytometry Luminex instruments. This dual strategy optimizes the testing process flow and serves as a contingency in the event of supply issues.

HPA genotyping is performed on maternal, paternal and neonatal samples to determine potential incompatibility in HPA expression.

In this study, we sought to describe the frequency of FNAIT investigations referred to NPIRL over an 11‐year period, the proportion of cases with detectable maternal HPA antibodies and the distribution of the antibody specificities. By comparing the observed testing patterns with the expected disease incidence based on population data, this analysis provides evidence that FNAIT is substantially under‐identified in the Canadian population and underscores the need for improved awareness, standardized diagnostic pathways and consideration of earlier identification strategies.

## MATERIALS AND METHODS

We searched our electronic databases to identify patients investigated for FNAIT between 20 January 2014 and 31 December 2024. Patient samples submitted for investigation for other platelet‐related disorders, including platelet transfusion refractoriness, were excluded. The total number of samples, as well as associated dates of collection and testing, were captured. Samples were categorized by source (maternal, paternal or neonatal). Test results were reviewed to determine the presence and specificity of the detected HPA antibodies.

Genotyping of HPA antigens prior to 2017 was performed using a sequence‐specific primer PCR (SSP‐PCR) assay (ThromboType, Werfen, Barcelona, Spain). After 2017, testing is being performed using a microarray‐based assay (BeadChip, Werfen). These assays are designed to detect common, clinically significant single nucleotide polymorphisms encoding platelet glycoprotein antigens, including HPA‐1–6, ‐9, ‐11 and ‐15.

HPA antibody testing was performed using a multiplex bead‐based immunoassay (PakPlus and PakLx, Werfen) based on Luminex xMAP technology. Inconclusive or positive results were tested using a lab‐developed MAIPA assay [9]. In addition, incompatibilities at HPA‐15 between maternal and paternal/neonatal samples were reflexed for MAIPA CD109 testing to test for possible anti‐HPA‐15 antibodies.

All tests were performed in accordance with the manufacturer's general instructions and validated laboratory protocols.

This study did not involve collection of additional information beyond that available at the time of patient testing, and no identifiers or individually identifiable data are presented. Therefore, according to standard assessments by the Research and Ethics Board, this study did not require ethics approval.

## RESULTS

Complete FNAIT investigations included 1986 samples over the 11‐year study period. This included 1076 maternal, 620 paternal and 290 associated neonatal samples (Table 1). Median maternal age at the time of investigation was 31.7 years.

**TABLE 1:** Number of positive cases of foetal and neonatal alloimmune thrombocytopaenia per province/territory over the 11‐year study period compared to expected number of annual cases based on birth rate.

	Maternal samples	Positive cases of FNAIT	Total live births (2014–2024)	Expected number of annual cases of FNAIT based on 1:1000 prevalence	Current incidence rate
British Columbia	290	35	479,000	479	1:13,686
Alberta*	2	1	571,491	571	N/A
Saskatchewan	62	6	161,011	161	1:26,835
Manitoba	116	17	185,039	185	1:10,885
Ontario*	506	60	1,556,657	1557	1:25,944
Nova Scotia	55	5	88,515	89	1:17,703
New Brunswick	28	2	70,938	71	1:35,469
Prince Edward Island (PEI)	3	0	14,692	15	N/A
Newfoundland and Labrador	12	4	43,051	43	1:10,763
Yukon	2	0	4432	4	N/A
Northwest Territories (NWT)	0	0	7147	7	N/A
Nunavut	0	0	4674	5	N/A
Total	1076	130			

*Note*: Provinces marked by asterisks (*) also have a separate in‐province platelet immunology laboratory. Incidence rates were not calculated for Alberta, PEI, Yukon, NWT and Nunavut because of low number of maternal samples received.

Abbreviation: N/A, not applicable.

**FIGURE 1:**
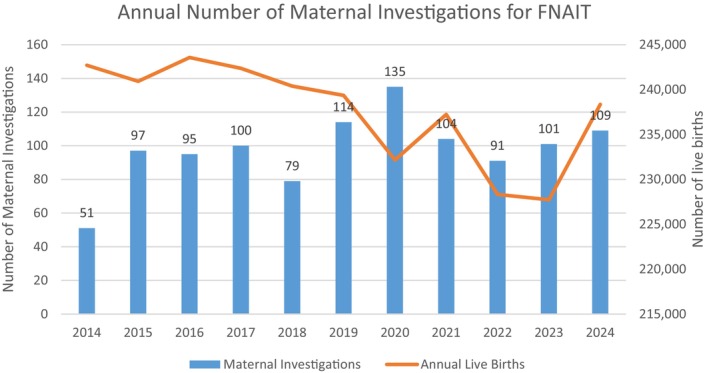
Annual number of foetal and neonatal alloimmune thrombocytopaenia (FNAIT) investigations at National Platelet Immunology Reference Laboratory (NPIRL) compared to annual live birth in Canada. Annual live births exclude live births from the provinces of Quebec and Alberta.

One‐hundred and thirty‐five HPA antibodies were detected in 130/1076 (12.1%) maternal investigations. Five patients had both HPA‐1a and HPA‐5b antibodies. HPA antibodies detected per year ranged from 7 to 21. There were no cases of anti‐CD36 detected during the study period. The total number of live births per province during the study period was calculated from annual data reported by Statistics Canada [6]. Estimated expected number of cases and current incidence rates were calculated using a prevalence of 1/1000 live births. Based on the current number of positive cases, the incidence of FNAIT in Canada (excluding the provinces of Alberta and Quebec) is 1/24,500. While the total number of samples received annually has increased over time, the change did not correlate with the annual number of live births in Canada (Figure 1).

HPA genotyping was performed on 1960 individual maternal, paternal and neonatal samples in the study period. Genotyping was routinely performed to determine the potential for incompatibility between maternal and paternal and/or neonatal specimens. Genotyping was not performed on 26 maternal samples because they had been genotyped in previous investigations. If both paternal and neonatal samples were received, the neonatal sample was prioritized when determining compatibility with the mother.

There were 730 family investigations (440 maternal and paternal only, 110 maternal and neonatal only and 180 with maternal, paternal and neonatal). Three‐hundred and forty‐six cases involved maternal investigation only.

Of the 130 cases with antibodies detected, 98 had family investigations performed. As expected, maternal–paternal and maternal–neonatal HPA incompatibility was strongly dependent on the maternal homozygous genotype (Table 2). Mothers homozygous for low‐frequency antigens showed the highest rates of paternal and neonatal incompatibility and were also more likely to have detectable HPA antibodies. This was most pronounced for HPA‐1, where 60.6% of HPA‐1b1b mothers were incompatible with paternal samples and 41.3% with neonatal samples, compared with 11.2% and 2.9%, respectively, for HPA‐1a1a mothers. Antibody positivity was markedly enriched among HPA‐1b1b mothers (75.0%), while it was low among HPA‐1a1a mothers (0.3%).

**TABLE 2:** Maternal genotyping results, antibody prevalence and incompatibility rates.

Genotype	Maternal cases	Relevant antibody, *n* (%)	Paternal incompatibility, *n* (%)	Neonatal incompatibility, *n* (%)
1a1a	748	2 (0.3%)	84 (11.2%)	22 (2.9%)
1b1b	104	78 (75.0%)	63 (60.6%)	43 (41.3%)
2a2a	873	1 (0.1%)	83 (9.5%)	27 (3.1%)
2b2b	17	0 (0%)	7 (41.2%)	3 (17.6%)
3a3a	434	0 (0%)	152 (35.0%)	44 (10.1%)
3b3b	161	5 (3.1%)	95 (59.0%)	32 (19.9%)
4a4a	1061	2 (0.2%)	2 (0.2%)	1 (0.1%)
5a5a	901	36 (4.0%)	87 (9.7%)	21 (2.3%)
5b5b	20	7 (35.0%)	12 (60.0%)	5 (25.0%)
6a6a	1057	0 (0%)	5 (0.5%)	1 (0.1%)
15a15a	296	13 (4.4%)	126 (42.6%)	38 (12.8%)
15b15b	239	0 (0%)	95 (39.7%)	37 (15.5%)

For HPA‐5, maternal–paternal incompatibility and antibody prevalence showed a pattern intermediate between highly immunogenic and low‐risk systems. Among HPA‐5a5a mothers, paternal incompatibility was observed in 9.7% of cases and neonatal incompatibility in 2.3%, with an antibody positivity rate of 11.9%. In contrast, HPA‐5b5b mothers demonstrated substantially higher incompatibility rates, with 60.0% incompatible with paternal samples and 25.0% incompatible with neonatal samples. Antibody positivity among HPA‐5b5b mothers was correspondingly elevated at 35.0%. These findings are consistent with the lower population frequency of HPA‐5b and support HPA‐5 as a clinically relevant but less immunogenic system than HPA‐1, characterized by marked enrichment of incompatibility among HPA‐5b5b mothers but a lower overall rate of alloimmunization.

For HPA‐3, both homozygous genotypes exhibited substantial incompatibility, with paternal incompatibility rates of 35.0% for HPA‐3a3a and 59.0% for HPA‐3b3b mothers, and neonatal incompatibility rates of 10.1% and 19.9%, respectively. Despite this high genetic mismatch, antibody prevalence was low (3.1% for HPA‐3b3b), indicating that incompatibility alone does not predict alloimmunization for this system.

HPA‐15 showed substantial incompatibility for both homozygous genotypes, with paternal incompatibility rates of 42.6% for 15a15a and 39.7% for 15b15b mothers and neonatal incompatibility rates of 12.8% and 15.5%, respectively. In contrast, incompatibility was rare for highly conserved systems such as HPA‐4 and HPA‐6, with paternal incompatibility rates below 1%.

Across all systems, neonatal incompatibility rates were consistently lower than paternal incompatibility rates, consistent with Mendelian inheritance. These data demonstrate that background genetic incompatibility is common for several HPA systems, particularly HPA‐1, ‐3, ‐5 and ‐15, but varies markedly by maternal genotype.

## DISCUSSION

This study provides the first national overview of referrals for FNAIT investigations to the Canadian NPIRL and demonstrates a substantial discrepancy between the expected incidence of FNAIT and the number of cases investigated in Canada. When compared with published population‐based estimates of 1 in 1000–2000 live births, the observed investigation rates suggest that FNAIT is under‐identified in the Canadian population.

Over the 11‐year period of analysis, 1076 maternal samples were submitted for FNAIT investigation. Although the number of investigations increased modestly over time (Figure 1), which may be a reflection of greater awareness and access to specialized testing, referral volumes remain far below what would be anticipated if FNAIT were consistently recognized and investigated.

Several factors likely contribute to this under‐identification. First, FNAIT frequently presents with mild or transient neonatal thrombocytopaenia that may resolve spontaneously without further investigation. In the absence of routine antenatal or targeted screening programmes, such cases are unlikely to be recognized. Second, neonatal thrombocytopaenia has a broad differential diagnosis, and the presence of alternative risk factors, such as prematurity, sepsis or maternal conditions, may preclude consideration of FNAIT. Third, severe cases may result in intrauterine foetal demise or early neonatal death before a diagnostic work‐up is initiated. If post‐mortem investigations are not performed or do not specifically assess platelet‐mediated mechanisms, FNAIT may remain undiagnosed. Finally, variability in clinician awareness and familiarity with diagnostic guidelines may further limit appropriate referral for laboratory investigation.

In this study, the case positivity rate among maternal samples investigated was 12.1%. Distribution of antibody specificity revealed anti‐HPA‐1a as the most common antibody, in keeping with published reports on the incidence of FNAIT in primarily White populations. While we provide the ability to capture ethnicity on the NPIRL referral forms, this information is rarely received on the requests for investigation and we cannot comment on the diversity within our testing population. Antibody detection assays performed at NPIRL are intended to identify all HPA antibodies clinically associated with FNAIT (HPA‐1a/1b, ‐2a/2b, ‐3a/3b, ‐4a/4b, ‐5a/5b, ‐6a/6b, ‐15a/15b and ‐CD36).

Family‐based investigations were incomplete in a proportion of cases, with many referrals involving maternal samples alone. While maternal antibody detection is informative, the absence of paternal or neonatal samples limits definitive assessment of incompatibility and foetal risk. Notably, instances of discordant genotyping between paternal and neonatal samples emphasize the potential limitations of relying solely on paternal genotyping for risk assessment and reinforce the importance of neonatal testing when available. These findings further illustrate the challenges of fully characterizing FNAIT in a referral‐based diagnostic model.

As the national platelet reference laboratory, it is expected that NPIRL receives the majority of requests for FNAIT investigations within Canada. There are, however, two additional provincial platelet testing labs located in Ontario and Alberta. Over a course of 30 years, one of these labs tested 1348 samples and identified 167 cases of FNAIT [10]. This finding of similarly low case numbers over several decades reinforces the conclusion that FNAIT is under‐identified nationally rather than simply underreported by a single laboratory. Comparable under‐recognition has been described in other countries lacking routine screening programmes, including Norway, prior to the implementation of structured antenatal strategies [11].

This study has several strengths, including a large national dataset spanning more than a decade and comprehensive laboratory assessment using validated immunological and molecular methods. The centralized nature of NPIRL testing allows for evaluation of referral patterns across most of Canada and provides valuable insight into current diagnostic practices.

Limitations include the absence of data on the ethnicity of the tested individuals and the lack of clinical outcomes data related to the presence of the maternal HPA antibodies. In addition, HLA‐DRB3*01:01 typing was not routinely performed, preventing evaluation of genetic susceptibility to anti‐HPA‐1a formation within the Canadian population.

While antibody testing assays performed by NPIRL are intended to identify all clinically significant HPA antibodies, it is possible that positive cases may have been missed. PakLx is a Luminex‐based screening assay routinely used by NPIRL to detect the common HPA antibodies and anti‐CD36. Samples with a positive anti‐CD36 result would be referred to an external laboratory for confirmatory testing. Incompatibility between maternal and/or paternal/neonatal for HPA‐15 is assessed in‐house with CD109 MAIPA. HPA genotyping using the HPA BeadChip kits was implemented in 2017 at NPIRL. This molecular assay detects 22 platelet antigens including HPA‐7, ‐8, ‐9 and ‐11 but was not included in this study because of their absence in the overall review period.

Other limitations include the lack of clinical correlation between maternal–foetal incompatibility with HPA antibodies and the presence of adverse outcomes in the foetus or newborn. Mothers without the HLA‐DRB3*01:01 antigen are less likely to form anti‐HPA‐1a antibodies [1, 11], the most common HPA antibody identified in this dataset. Since physicians did not routinely request this particular HLA genotyping for FNAIT investigations, the population frequency of HLA DRB3*01:01 in Canada could not be determined. This study could not distinguish between investigations sent because of affected pregnancies and those sent because of a positive family history rather than an index patient history. Current guidelines recommend investigation for HPA antibodies in the setting of clinical symptoms including neonatal thrombocytopaenia as well as in sisters of previously affected individuals [5].

In conclusion, FNAIT is a clinically significant pregnancy complication that is associated with increased morbidity and mortality to the foetus and neonate. In this national analysis of laboratory investigations at the Canadian NPIRL, the number of referrals and antibody positive cases identified over an 11‐year period was significantly lower than expected based on published incidence rates and Canadian live birth statistics.

Although NPIRL is able to perform comprehensive laboratory testing to assess for maternal alloimmunization, the current diagnostic pathways rely on clinical presentation and clinician‐initiated testing. As a result, cases with minimal clinical symptoms and cases associated with foetal or early neonatal death are likely to have been missed.

Improved recognition of FNAIT through increased clinician awareness, standardized national investigation algorithms and routine antenatal screening may help reduce missed diagnoses and improve clinical management. These findings highlight opportunities to re‐assess and improve our current diagnostic approaches to FNAIT in Canada.

## CONFLICT OF INTEREST STATEMENT

The authors declare no conflicts of interest.

## Data Availability

The data that support the findings of this study are available on request from the corresponding author. The data are not publicly available due to privacy or ethical restrictions.
